# Supplementary material for: Reciprocity of social influence
Source: Nat Commun. 2018 Jun 26;9:2474. doi: 10.1038/s41467-018-04925-y (PMC6018808; doi:10.1038/s41467-018-04925-y)
Supplement: Supplementary file 1 — Supplementary Information [file 41467_2018_4925_MOESM1_ESM.pdf]

Supplementary Information

Reciprocity of social influence

Mahmoodi et al.

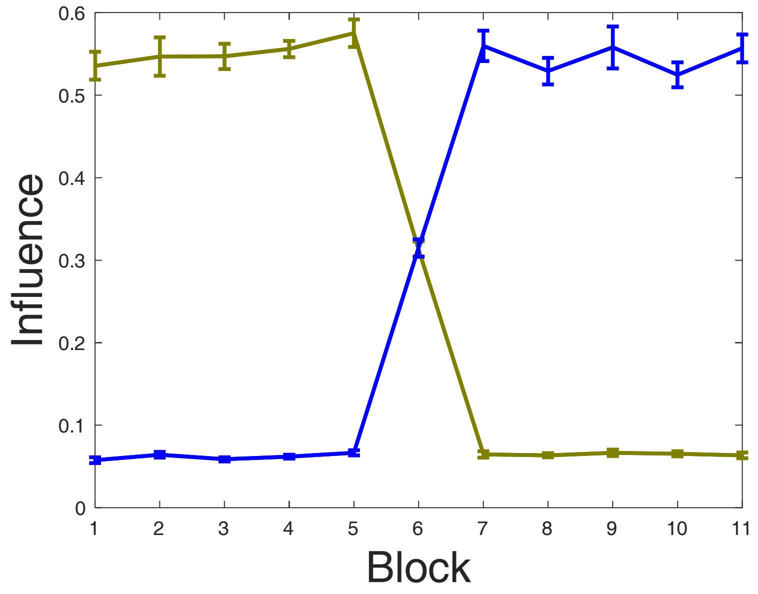

Supplementary Figure 1: Amount of influence that the virtual partner took from subjects in experiment 2, averaged across all participants. Error bars depict the standard error of the mean across participants. Error bars indicates s.e.m.

Supplementary Note 1:

Effect of actual and perceived performance and liking of the partner on influence in experiment 1: To rule out various potential confounds (perceived and actual precision of partners across conditions, participants' precision and liking of the partner across conditions) we applied the following linear mixed model:

$$I = \beta_{1s} + \beta_2 \times Cond + \beta_3 \times P_{self} + \beta_4 \times P_{partner} + \beta_5 \times PerceivedP_{partner} + \beta_6 \times like_{partner} (1)$$

I is the average influence of the partner on the participant in a specific condition, $Cond$ is the experimental condition (baseline, susceptible, insusceptible), P_{self} is the actual precision of the participant, $P_{partner}$ is the actual precision of the partner, $PerceivedP_{partner}$ is the performance rating, i.e. the perceived performance of the partner, and $like_{partner}$ is how much the participant liked the partner. To account for participant specific baseline influence we let the intercept coefficient vary across participants by including random effects of the form $\beta_{1s} = \beta_{10} + b_{1s}$ where $b_{1s} \sim N(0, \sigma^2)$ and s is the participant id. All other factors were fixed effects.

We found a significant effect of condition (t(45) = 3.59, p = .0007) on influence while the actual precision of the participants (t(45) = -.38 p = .7), the actual partners' precisions (t(45) = -1.89 p = .06), the performance rating for the partners (t(45) = 1.51 p = .13), and the like rating for partners (t(45) = .43 p = .66) had no significant impact on influence.

We also did not find any correlation between the difference in perceived performance and the difference in influence across participants (Pearson correlation coefficient, between susceptible and baseline, r = .19 p = .48, between susceptible and insusceptible r = .2 p = .45).

Moreover, using a repeated measure ANOVA we found that there wasn't any difference neither in the precision of the partners (F(1.7,27) = 1.06, p = .35) nor in the precision of the participants (F(1.77,28.34)

= .31, p = .7) across different conditions. Participants' precision after the second estimate didn't vary across conditions either (repeated measures ANOVA, F(1.97,31) = .39, p =.67).

Effect of confidence on influence in experiment 1:

To scrutinize the interaction between confidence and condition on influence, we used a linear mixed model with the influence of the partner on the participant as the dependent variable and the condition (baseline, susceptible or insusceptible) and participant's confidence as independent variables:

$$I = \beta_{1s} + \beta_{2s} \times Cond + \beta_{3s} \times C + \beta_{4s} \times C * Cond \quad (2)$$

I is the influence of the partner on the participant, $Cond$ is the condition, and C is participant's confidence. We categorised our confidences into three levels: low (confidence 1 and 2), intermediate (confidences 3 and 4), and high (confidences 5 and 6). Then for each category we computed the average influence for that category.

The intercept (β_{1s}) and all slopes ($\beta_{2s}, \beta_{3s}, \beta_{4s}$) were allowed to vary across participants by including random effects of the form $\beta_{ks} = \beta_{k0} + b_{ks}$ where $b_{ks} \sim N(0, \sigma^2)$. We first compared the susceptible to the baseline condition. While the effect of confidence and intercept were significant (β_{10} t(964) = 10.22 p = 0, β_{30} t(96) = -5 p = 2×10^{-6}) there was no effect of condition (β_{20} t(96) = 1.36 p = $.17$) nor of the interaction between condition and confidence (β_{40} t(96) = -.59 p =.55). Since the effect of the interaction term on the influence was the weakest factor, we removed it from the model to reduce the number of model parameters and therefore increase the statistical power of the remaining model. As a consequence we obtained a significant effect of condition for the model without interaction term (β_{20} t(97) = 2.43 p = $.01$).

We then compared the susceptible condition to the insusceptible condition. All four factors of the complete model (eq. 2) were significant (β_{10} t(96) = 9.45 p = 2×10^{-1} , β_{20} t(96) = -5.75 p = 1×10^{-7}, β_{30} t(96) = -5.78 9×10^{-8}, β_{40} t(96) = 5. 15 p = 1×10^{-6}).

Effect of confidence on influence in experiment 2A:

We applied the same procedure as above to rule out the effect of confidence on influence in experiment 2A:

$$I = \beta_{1s} + \beta_{2s} \times Cond + \beta_{3s} \times C + \beta_{4s} \times C * Cond \quad (3)$$

where I is the influence of the partner on the participant for each confidence category, $Cond$ is the condition of the experiment (susceptible, insusceptible) and C the confidence category. The intercept (β_{1s}) and all slopes ($\beta_{2s}, \beta_{3s}, \beta_{4s}$) were allowed to vary across participants by including random effects of the form $\beta_{ks} = \beta_{k0} + b_{ks}$ where $b_{ks} \sim N(0, \sigma^2)$. While the effect of intercept and confidence were significant (β_{10} t(167) = 6.75 p = 2×10^{-10}, β_{30} t(167) = -3.15 p = $.001$) there was no effect of condition (β_{20} t(167) = 1.4 p = $.16$) nor of the interaction between condition and confidence (β_{40} t(167) = -.15 p =.87). Since the effect of the interaction term on the influence was the weakest factor, we removed it from the model to reduce the number of model parameters and therefore increase the statistical power of the remaining model. As a consequence we obtained a significant effect of condition for the model without interaction term (β_{20} t(168) = 335 p = $.0009$).

Effect of perceived performance on influence in experiment 2A:

We constructed a linear mixed model

$$I = \beta_{1s} + \beta_2 \times Cond + \beta_3 \times P_{self} + \beta_4 \times P_{partner} \qquad (4)$$

where *I* is the average influence of the partner on the participant in a specific condition, $Cond$ is the experimental condition (susceptible, insusceptible), P_{self} is the performance rating for self and $P_{partner}$ is the performance rating for the partner. We included random effects to allow for participant specific intercepts ($\beta_{1s} = \beta_{10} + b_{1s}$ where $b_{1s} \sim N(0, \sigma^2)$ while all other factors were fixed effects. While there was a significant effect of condition (t(56) = -4.1 p = .0001) , neither the performance rating for self (t(56) = -.04 p = .3) nor for the partner (t(56) = .16 p = .87) had a statistically significant impact on influence.

Choosing random or fixed effects:

In all the above models, we included random effects in the intercept to allow for a participant specific baseline influence. In models (2) and (4) we additionally included random effects in the other coefficients to account for repeated measurements from individual participants.

Potential confound of the gender of the experimenter in experiment 2A and 2B:

One could argue that the difference in reciprocity between playing with an alleged human or computer partner could be a confound of the gender of the experimenter. In our experiments, we randomly assigned male and female experimenter to participants. To rule out an effect of the gender of the experimenter, we compared the reciprocity between the participants which were assigned male and female observers in experiments 2A and 2B. Neither for alleged human nor for computer partners a significant effect of the experimenter's gender was found (Wilcoxon rank sum test, experiment 2A p = .62, Z = .48, experiment 2B p = .93, Z= -.07).

Effect of distance of the initial estimates on influence and reciprocity:

To answer this question, we first excluded all trials during which the participant reported a high confidence (confidence levels 5 and 6) as in these trials the estimate of the partner was always around our participants' estimates by construction (see Methods). We then divided our trials into 4 categories depending on the initial distance between the participants' and their partner's estimates. These 4 categories were defined by the four intervals [0°;45°), [45°;90°), [90°;135°) and [135°;180°) which together spanned the whole range of possible distances. To investigate the effect of distance on influence we then conducted a mixed ANOVA with four distance categories and two conditions as within subjects factors and experiment (2A or 2B) as between subjects factor. We did not find an effect of distance (F(2.6,151) = 1.17 p = .31) nor condition (susceptible or insusceptible) (F(1,58) = 2.76 p = .1). We also did not find a significant interaction between distance and condition (F(2.6,151) = 1.47 p

= .22), nor between distance, condition and experiment (F(2.6,151) = 1 p = .38). The interaction between distance and experiment was around the significance threshold (F(2.6,151) = 2.7 p = .05). And finally, in line with our previous finding, there was a significant interaction of experiment and condition (F(1,58) = 4.36 p = .04).

Next we investigated the effect of distance on reciprocity in experiment 2A. We calculated the reciprocity for each distance category and each participant and a repeated measure ANOVA revealed no effect of distance on reciprocity (F(2.61,75) = 1.63 p = .16). This analysis was only carried out for experiment 2A and not 2B as there was no reciprocity observed in experiment 2B.

Effect of partner's susceptibility on performance rating separately for experiment 2A and 2B:

We computed the difference between the participants' ratings of their own performance in the susceptible and the insusceptible condition (Supplementary Figure 2). There was no difference between experiments 2A and 2B (Wilcoxon rank sum test Z = .52 P = .6). We therefore aggregated the data from both experiments and found a significant difference between the performance rating in the susceptible and insusceptible conditions (Figure 3E, Wilcoxon sign rank test, Z = 3.04 p = .002). For experiment 2A alone the difference between the conditions was just above significance threshold (Supplementary Figure 2, panel A Wilcoxon sign rank test Z=1.86, p=0.06), while for experiment 2B alone the effect was significant (Supplementary Figure 2, panel B, Wilcoxon sign rank test Z = 2.46 P = .01).

We repeated the above procedure for the participants' ratings of their partners' performance. There was no difference between experiments 2A and 2B (Wilcoxon rank sum test, Z = -.47 P = .63). We therefore aggregated the data from both experiments and found no significant difference between the performance rating in the susceptible and insusceptible conditions (Wilcoxon sign rank test, Z = -.9 p = .36).

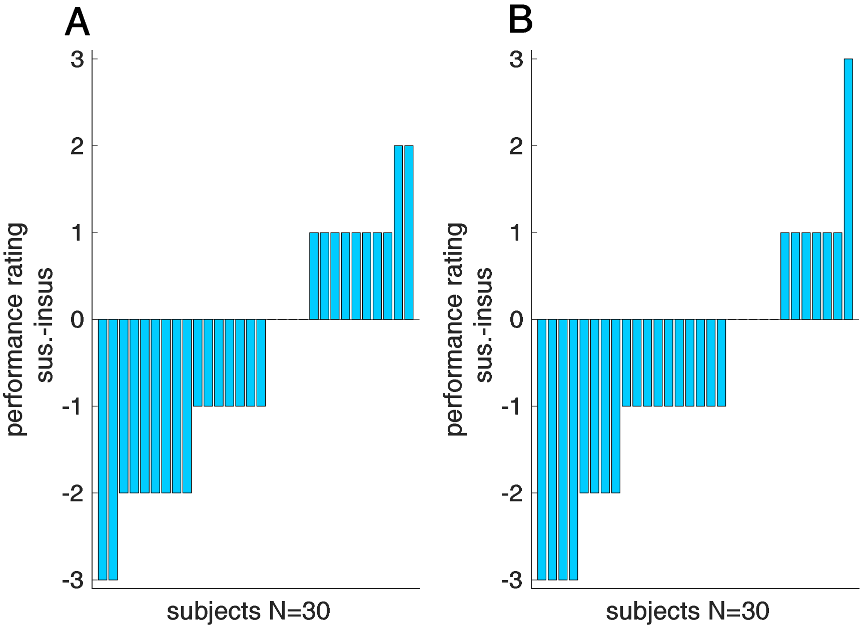

Supplementary Figure 2*:* Participants' rating of their own performance in the insusceptible minus the susceptible condition separately for experiment 2A (A) and 2B (B).

Experiment 1 without excluding any participant:

For the results presented in the main part three participants were excluded from the analysis of experiment 1. Two of them got away from their own and their partners' initial estimates in many trials and therefore violated the task instructions. The third one did not pay attention during the experiment and during the debriefing we realised that the participant did not understand the differences between the partners. Here, we reanalysed experiment 1 using the data from all participants including these three participants that were originally excluded. Our results show that reciprocity was still significantly above zero in both cases (Supplementary Figure 3; Wilcoxon sign rank test, baseline vs susceptible z = -2.5 p = .01, and insusceptible vs susceptible z = -3.47 p =.0005).

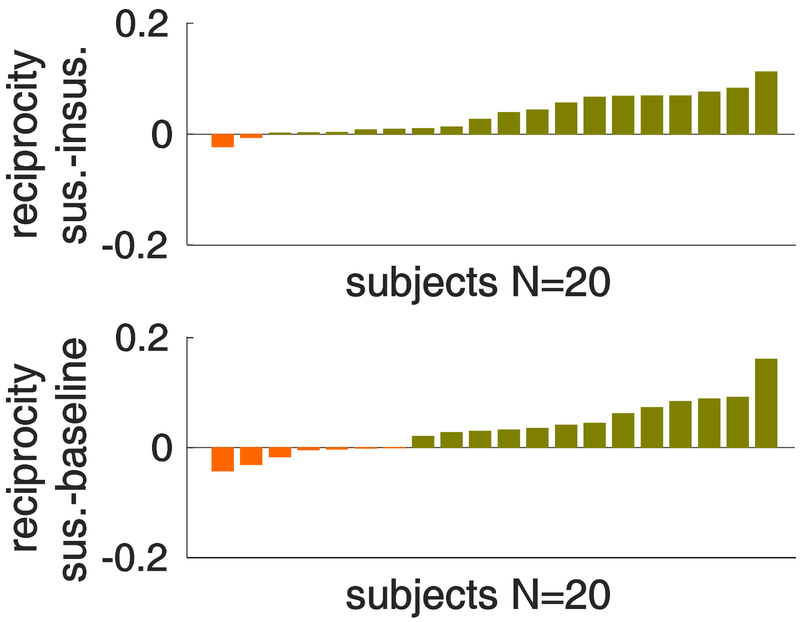

Supplementary Figure 3: Results of experiment 1 including the three participants which were excluded in the analysis presented in the main text.
